# The Interior of the Urethra Viewed by a Magnesium Light

**Published:** 1867-04

**Authors:** E. Andrews

**Affiliations:** Professor of Surgery in Chicago Med. College


ARTICLE XIX.

### THE INTERIOR OF THE URETHRA VIEWED BY A MAGNESIUM LIGHT.

By E. ANDREWS, A.M., M.D., Prof. of Surgery in Chicago Med. College.

The invention of the endoscope, simultaneously by a French and by a Dublin surgeon, has opened a new field, both in the pathology and treatment of the urethra. The endoscope consists of a lamp, a perforated mirror, and an urethral tube. These, when combined, throw a condensed light into the urethra, and enable the surgeon to inspect every part of it. One of the important fruits of this instrument is, the discovery that the chronic inflammation remaining after certain cases of gonorrhœa is granular in its character, and is, in fact, the same disease as granular conjunctivitis, granular laryngitis, and granular inflammation of the cervix uteri.

Some months ago, I had an endoscope constructed after the Parisian plan, and used it with some degree of satisfaction; but there is often a deficiency of light in these instruments, rendering the view unsatisfactory, unless all parts are in perfect order. Seeking to overcome this evil, I one day procured some small magnesium wire, which, when held in the flame of a lamp, burns with a white light, whose brilliancy dazzles like the glare of the sun at noonday. Introducing the endoscope into the urethra of a patient, I caused a friend to insert the wire into the flame of the lamp. The result was to illuminate the urethra magnificently. The mucous membrane, with every little fold or patch of varied color, was as plainly in view as could possibly be desired. It could not have been seen any better, had it been dissected and laid in the sunlight. By gradually withdrawing the tube, the whole of the canal may successively be seen as it collapses across the end of the tube. Seeing the perfection of this illumination, I have ordered a spring and some small wheel-work attached to the lamp, so that the wire may be made to advance into the flame without the help of an assistant. In this way, no doubt, the difficulty of the illumination will be fully overcome, and the urethra can be inspected almost as easily, and quite as perfectly, as the tongue.

